# Water-triggered in-situ gelling phospholipid oil enema for enhanced budesonide delivery and mucosal healing in ulcerative colitis

**DOI:** 10.1016/j.ijpx.2026.100647

**Published:** 2026-08-21

**Authors:** Ting Ouyang, Yumo Chen, Yiying Jia, Jiarui Li, Zhouyang Tan, Kaili Lu, Minmin Wang, Helin Xu, Lifen Wang

**Affiliations:** a School of Chinese Medicine, Wenzhou Medical University, Wenzhou City, Zhejiang Province 325035, China; b Department of Pharmaceutics, School of Pharmaceutical Sciences, Wenzhou Medical University, Wenzhou City, Zhejiang Province 325035, China; c Department of Gastrointestinal Surgery Nursing Unit, Ward 442, The First Affiliated Hospital of Wenzhou Medical University, Wenzhou City, Zhejiang Province 325000, China; d Hainan Pharmaceutical Research and Development Science Park, Hainan Medical University, Haikou 571199, China

**Keywords:** Ulcerative colitis, Budesonide, Phospholipid oil, Water-responsive, Mucosal healing

## Abstract

Budesonide (BUD) enema therapy for ulcerative colitis (UC) is limited by poor solubility, inadequate bio-adhesion, and rapid clearance due to intestinal peristalsis. To address the limitations of conventional budesonide enemas, we designed a water-triggered in situ phase-transition phospholipid formulation (termed PG oil). This system comprises soybean phosphatidylcholine (PC-98), glyceryl dioleate (GDO), propylene glycol, and anhydrous ethanol, and achieves markedly enhanced BUD solubilization (35 mg/mL), in contrast to its negligible aqueous solubility (0.021 mg/mL). Upon contact with colonic fluid, PG oil rapidly underwent sol-gel transition, forming a bio-adhesive lamellar liquid crystalline gel that serves as both a physical mucosal barrier and a sustained-release drug depot. In a dextran sulfate sodium (DSS)-induced colitis mouse model, rectal administration of BUD-PG oil (0.3 mg/kg) significantly outperformed free BUD suspension, as evidenced by restored body weight, reduced disease activity index, normalized colon length, and decreased spleen index. Immunohistochemistry revealed marked suppression of pro-inflammatory cytokines (IL-6, TNF-α, IL-1β, MCP-1) in colonic tissue. Histological analysis demonstrated that BUD-PG promoted favorable mucosal repair characterized by reduced collagen deposition (Masson's trichrome: from 48.5% to 16.7%) while restoring gut barrier integrity through replenishment of goblet cells and upregulation of tight junction proteins (ZO-1, Occludin-1, Claudin-5, β-catenin). Collectively, this water-responsive in situ gelling phospholipid oil platform addresses critical limitations of conventional BUD enemas by combining sustained local drug delivery with physical mucosal protection, offering a promising therapeutic strategy for comprehensive mucosal healing in UC.

## Graphical abstract

**Figure:**
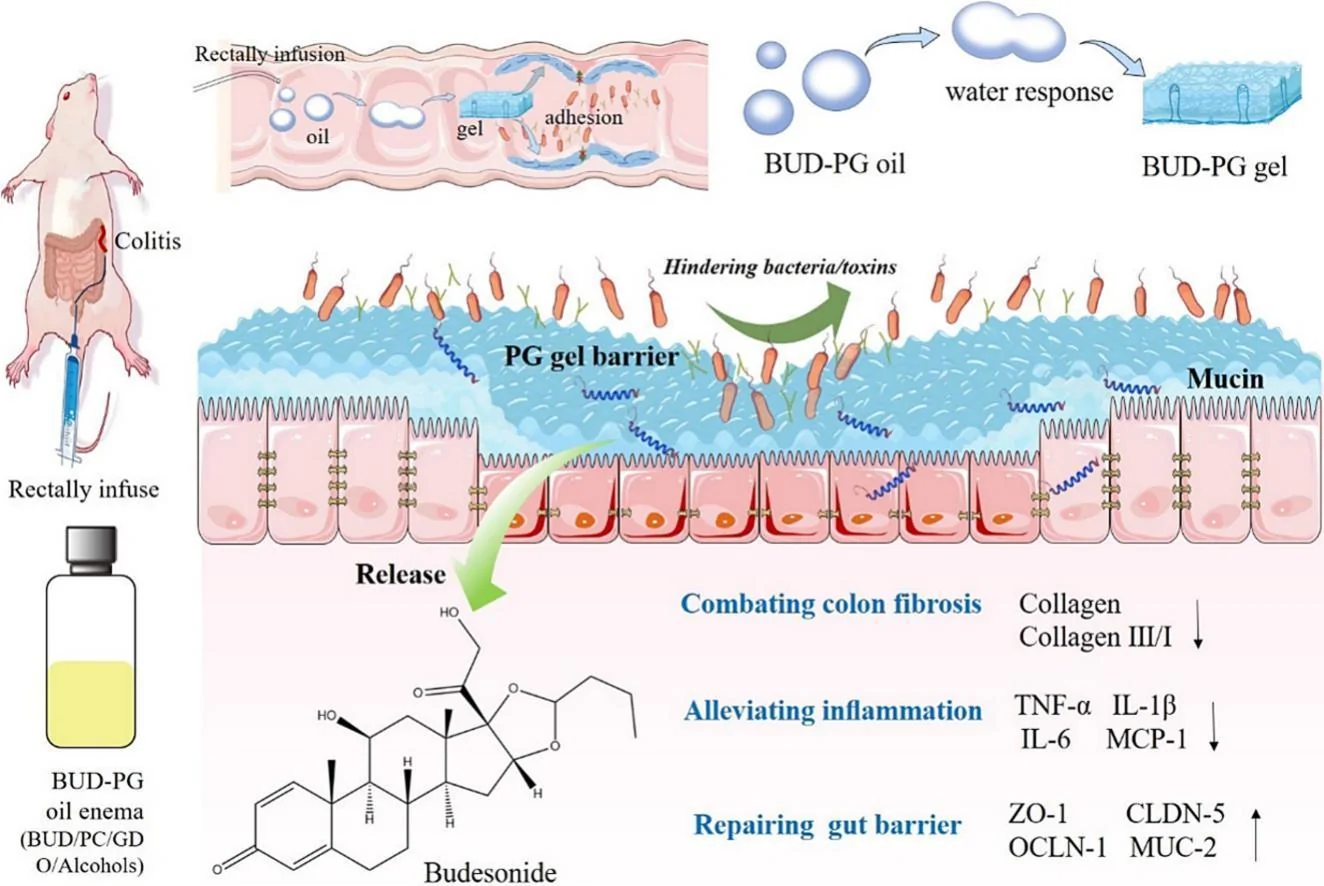

## 1 Introduction

Inflammatory bowel disease (IBD) is a chronic immune-mediated disorder affecting the gastrointestinal tract, characterized by non-specific and inflammatory features (Agrawal et al., 2025). Its precise etiology remains elusive, but it is now primarily understood as the result of complex interactions among genetic susceptibility, environmental triggers, intestinal microbiota dysbiosis, and dysregulated immune responses (Kaplan, 2025). IBD manifests in two primary forms: Crohn's disease (CD) and ulcerative colitis (UC) (Tian et al., 2024). Currently, UC has become a global disease, with its incidence rate in China also showing a rising trend. UC follows a recurrent course of flare-ups and remissions, characterized primarily by mucosal inflammation that begins distally and extends proximally in a continuous pattern, potentially involving the entire colon. The typical clinical symptoms of UC primarily include rectal bleeding, decreased stool consistency, and increased bowel movements (passing stool, mucus, and blood), along with abdominal pain, bloating, and bowel or bladder incontinence (Ungaro et al., 2017; Shivaji et al., 2020). These intestinal symptoms may be accompanied by systemic symptoms such as fatigue, fever, dehydration, loss of appetite, and weight loss. UC can significantly impact one's personal life, causing patients to suffer both physically and psychologically. Currently, there is no definitive cure for UC. The primary therapeutic goals are symptom relief, anti-inflammatory and analgesic effects, restoration of intestinal barrier structure and function, as well as prevention and management of complications to improve patients' quality of life. The first-line therapeutics typically involves 5-aminosalicylic acid (5-ASA), corticosteroids, immunosuppressants, and biologics (Chand et al., 2025; Deng et al., 2009; Zhang et al., 2024a).

Budesonide (BUD) is a potent corticosteroid with high topical anti-inflammatory activity and extensive first-pass hepatic metabolism (∼90%), making it suitable for localized treatment of intestinal inflammation (Münch et al., 2016). Unlike systemic corticosteroids, orally or rectally administrating BUD is indicated for the induction of remission in patients with mild-to-moderate UC, particularly for colitis localized to the distal colon and rectum. To reduce the first-pass hepatic metabolism, BUD has been developed as delayed- and extended-release tablets for colon-targeted release (Sun et al., 2018). Alternatively, BUD enema suspensions for distal colorectal disease offer superior efficacy and a better safety profile compared to oral counterparts (Ali et al., 2024). Nevertheless, their clinical utility is compromised by poor drug solubility and inadequate mucosal penetration, yielding remission rates below 40% even after 6–8 weeks of daily administration (Sandborn et al., 2015). To overcome these issues, mucus-penetrating budesonide nanosuspension has been developed as enema for inflammatory bowel disease, achieving the more obvious alleviation on the colorectal inflammation (Date et al., 2018). But this budesonide nanosized enema was confronted with poor colon adhesion and short retention in term of colitis-relevant diarrhea and colon peristalsis. It is imperative for enema formulation with capability of solubilizing hydrophobic drug, strong bio-adhesion and low toxicity to ensure safety and reliability.

A novel in situ gel-forming oil composed of soybean phosphatidyl choline, dioleoyl glycerol and ethanol/propylene glycol have been developed as enema vehicle of hydrophobic laquinimod for ulcerative colitis therapy (Zhao et al., 2023). This system was the oil-like liquid with low viscosity in vitro to solubilize hydrophobic drug, while it underwent in situ gelation on colon mucosa after rectal administration in response to the aqueous environments. Moreover, unlike most phospholipid-based injectable gel, in situ gel formation of this oil system is based on self-assembly of phospholipids together with dioleoyl glycerol, forming robust gel-like film on mucosal tissues (Zhang et al., 2024b). Moreover, as major component of cell membrane, phospholipids was reported to ameliorate mouse colitis via restoring colonic goblets (Wang et al., 2019).

**Fig. 1:**
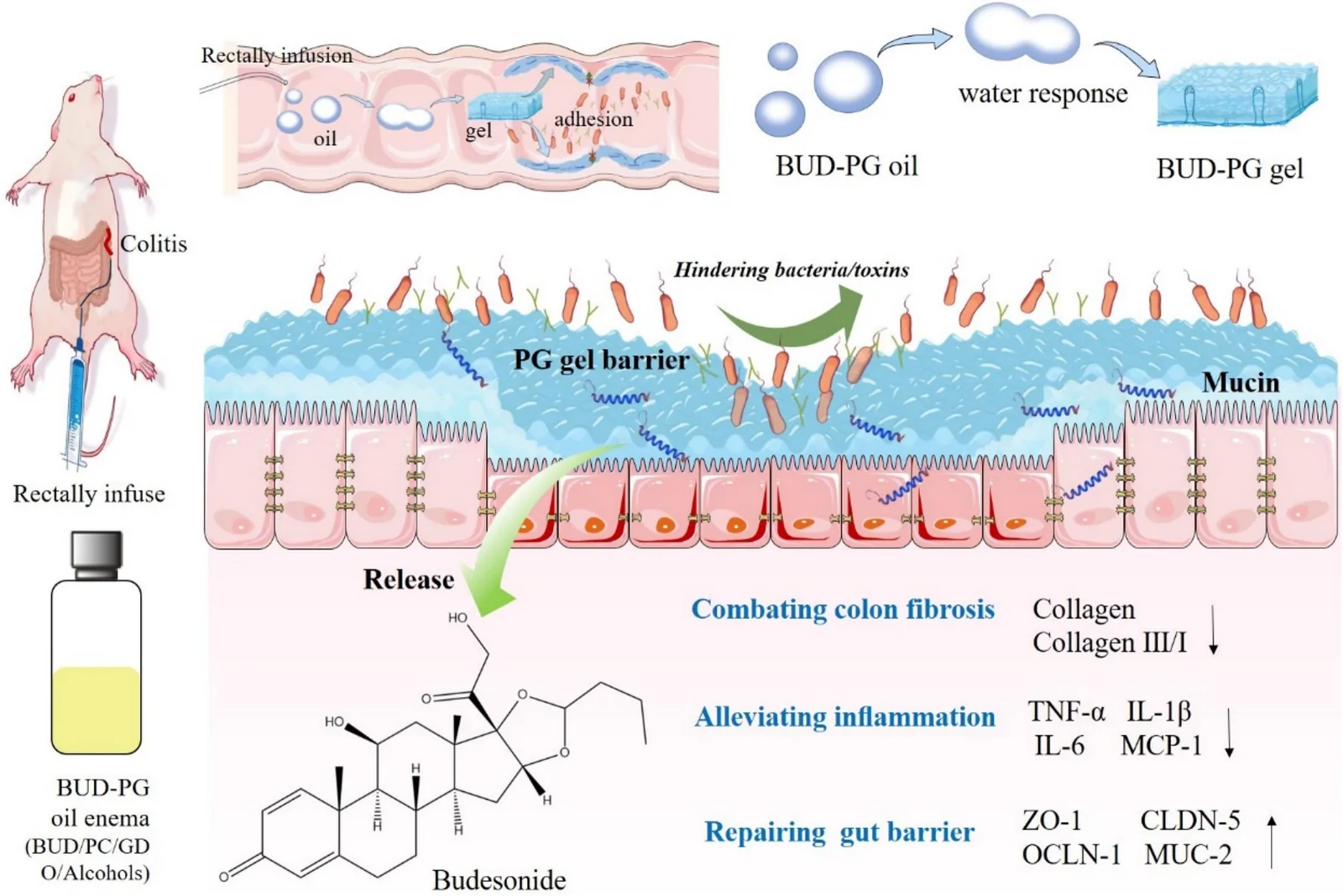
PG oil as enema to solubilize BUD for ulcerative colitis treatment.

In this study, in situ gel-forming oil, herein named PG oil has been developed as enema to deliver hydrophobic BUD for ulcerative colitis (Fig. 1). It was expected to overcome issues of low solubility of BUD and poor colon retention. After rectal administration, BUD-solubilizing PG oil (BUD-PG) undergoes phase separation in response to colonic fluid, adhering tightly to the colonic surface to form a gel film that served as a physical barrier. This barrier physically protected the damaged mucosal barrier by isolating bacteria and fecal flushing. Simultaneously, BUD were gradually released from the gel-like film to exert therapeutic effects. In this study, we studied water-response of PG oil and characterized its intrinsic morphology. Besides, the solubility of BUD in PG oil was also evaluated and its release profile from PG gel was also tested. Finally, the therapeutic efficacy of BUD-PG formulation was demonstrated in a mouse model of ulcerative colitis (UC) induced by dextran sulfate sodium (DSS), showing effective repair of colonic morphology, inhibition of colonic inflammatory responses, and restoration of the colonic epithelial mucosal barrier.

## 2 Materials and methods

### 2.1 Materials and regents

Soybean phosphatidyl choline (PC-98, purity of 98%) was purchased from Tianfeng Bio-Chem Co., Ltd. (Shenyang, China). Glyceryl dioleate (GDO) was obtained from Huaxiangkejie biochemical technology (Wuhan, China). Budesonide (BUD, purity of 98%) was purchased from Macklin company (Shanghai, China). Dextran sulfate sodium (DSS, 40 kDa) was purchased from Shanghai Yeasen Biotechnology Co., Ltd. (China). ELISA kits of IL-6, IL-1β, TNF-α and IL-10 were purchased from Shanghai Jianglai Co., Ltd. (China).

### 2.2 Preparation of PG oil

PG oil was prepared by the previously reported method with modifications (Zhang et al., 2024b). Briefly, soybean phosphatidylcholine (PC-98, 4 g, 98% purity) was first dispersed in anhydrous ethanol (1 mL) under stirring to form a gel. Glyceryl dioleate (GDO, 5 g) was then added gradually to dissolve the PC-98 gel, yielding a viscous oil solution. Finally, propylene glycol (1 mL) was incorporated to reduce the viscosity, producing a clear, yellowish oil. As the hydrophobic model drug, budesonide powder (35 mg) was directly dissolved in PG oil (1 g) under stirring until the clear solution was formed.

### 2.3 In situ sol-gel transition of PG oil

Distilled water was gradually added to PG oil (1 mL) under stirring to observe its appearance changes. The gel formation was confirmed by the vial tilting method. When no flow within 1 min was regarded as the gel formed after inverting the vial, the required amount of distilled water for gelling was recorded. Each test was repeated three times. Besides, PG oil was labeled by 3,3′-dioctadecyloxacarbocyanine perchlorates (DIO) to observe the morphology of PG gel. PG oil (500 μL) was spread on distilled water and the morphology of PG gel was observed by confocal laser scanning microscopy (CLSM). For BUD-loaded PG oil, the status of drug after gelling was also observed by CLSM.

### 2.4 The solubility of BUD in PG oil

BUD powder (100 mg) was suspended to PG oil (1 mL) and the oil suspension was stirred overnight. After centrifuging at speed of 5000 rpm for 30 min, the concentration of BUD in the supernatant oil was determined by using a spectrophotometer (TU1901, perking) at wavelength of 242 nm. As control, the solubility of BUD in distilled water was also detected. For UV spectrophotometric method, a standard calibration curve (C = 34.0138 A - 0.04750, *R* = 0.9997) was constructed using BUD standard solutions in acetonitrile at concentrations ranging from 2 to 50 μg/mL. This analytical method is specific, accurate, precise, and suitable for the intended purpose.

### 2.5 Contact angle test

To evaluate the hydrophilicity of PG oil, the sessile drop method was used by applying a drop of PG oil on the samples of various surfaces (glass plates, polypropylene plates or rat buccal mucosa). The images were captured by a camera and transferred to Image J software, version 1.51n (National Institute Health, New York, NY, USA) to measure the contact angle.

### 2.6 In vitro release of BUD from PG gel

In vitro release of BUD from PG gel was performed by the method in our previous study (Zhao et al., 2023). Briefly, a piece of filter paper (2 cm in diameter) was pre-wetted with distilled water, onto which 0.3 g of BUD-PG oil was loaded to allow gel formation. The resulting BUD-PG-laden filter paper was then placed at the bottom of a vessel, followed by the addition of 200 mL of release medium (pH 7.4 phosphate-buffered saline). The assembly was incubated in a thermostatic shaker at 37 °C with constant agitation at 100 rpm. At predetermined time points, 1 mL of the release medium was withdrawn, diluted with methanol, and analyzed by UV spectrophotometry. An equal volume of fresh medium was immediately replenished to maintain sink conditions. The cumulative percentage of BUD released was calculated as [M_t_/M_total_] × 100%, where denotes the amount of BUD released at time t, and M_total_ represents the total BUD initially loaded in the PG oil.

### 2.7 Animal experiments

Male ICR mice, aged 6–8 weeks and weighing 20–22 g at the start of the experiment, were obtained from the Experimental Animal Center of Wenzhou Medical University (specific pathogen-free grade). All animals were housed in groups of five per cage under controlled environmental conditions: temperature 22 ± 2 °C, relative humidity 50 ± 10%, and a 12-h light/dark cycle. Animals had ad libitum access to standard rodent chow and filtered water throughout the acclimatization period (7 days) and during the experiment, except for the DSS treatment period when DSS was provided in drinking water. A priori animal sample-size calculation was performed based on our preliminary data, with a power of 80%, a two-sided alpha of 0.05, and an expected effect size of 25% difference in colon length between treatment groups. This yielded a minimum requirement of 4 mice per group. To account for potential attrition, 5 mice per group (*n* = 5) were used in each experimental group, with a total of 25 mice across all groups (Control, BUD, PG, BUD-PG, and Normal). Health status was monitored daily by veterinary staff, and no animals exhibited signs of illness or distress prior to DSS administration. For the DSS-induced colitis model, animals were randomly allocated to experimental groups using a computer-generated randomization sequence by an investigator not involved in the subsequent treatments or outcome assessments. All investigators performing DAI scoring, histological evaluation, immunohistochemical quantification, and immunofluorescence analysis were blinded to group allocation. Animals that failed to complete the DSS treatment period due to severe morbidity (>25% body weight loss or signs of impending death) or technical issues during enema administration (e.g.*,* perforation or significant leakage) were to be excluded from analysis. All animal procedures were performed in strict compliance with the guidelines established by the National Animal Care and Use Committee and received approval from the Institutional Animal Committee (approval No. xmsq 2024–0281).

#### 2.7.1 Experimental regimen of BUD-PG oil on colitis mice

A total of 25 male ICR mice were randomly assigned to five experimental groups: control (DSS-induced colitis without treatment), BUD (colitis mice receiving BUD suspension), PG (colitis mice receiving PG oil alone), BUD-PG (colitis mice receiving BUD-PG oil), and normal (healthy mice receiving PBS). Colitis was induced by administering 4% dextran sodium sulfate (DSS) in drinking water for 7 consecutive days. On day 8 post-DSS initiation, mice in the treatment groups daily received either BUD suspension or BUD-PG oil for six days via rectal administration. All formulations were equilibrated to body temperature (37 °C) for 30 min prior to administration and gently vortexed to ensure homogeneity. The BUD dose was 0.3 mg/kg body weight, and the administration volume was 0.2 mL per mouse (approximately 100 μL per 10 g body weight), delivered using a 1-mL insulin syringe fitted with a flexible polyurethane catheter (0.7 mm outer diameter). For the administration procedure, mice were gently restrained in a vertical position with the tail elevated without anesthesia. A small amount of sterile lubricant (glycerol) was applied to the distal 5 mm of the catheter to minimize mucosal trauma. The catheter was then carefully inserted into the rectum to a standardized depth of 4 cm, which corresponds to the distal colon region where DSS-induced inflammation is most pronounced. The formulation was slowly infused over 15–20 s to avoid triggering the defecation reflex. After complete infusion, the catheter was gently withdrawn while the anal sphincter was lightly compressed with sterile gauze for 30 s to prevent reflux and ensure retention. Mice were then maintained in a head-down position at approximately 15° inclination for 5 min to facilitate formulation distribution throughout the distal colon and minimize leakage. For BUD-PG oil formulation, the drug-loaded PG oil was prepared freshly on each administration day and stored at room temperature in sealed amber vials protected from light until use. BUD suspension (control) was freshly prepared by dispersing an equivalent amount of BUD powder in 0.5% carboxymethylcellulose sodium (CMC—Na) solution, with continuous stirring for 10 min to maintain homogeneity, and was used within 1 h of preparation. As control, healthy mice received saline only. Throughout the experiment, body weight, stool consistency, and rectal bleeding were monitored daily, and the disease activity index (DAI) was calculated according to a previously established protocol (Zhao et al., 2023). All animals were humanely killed by carbon dioxide inhalation followed by cervical dislocation at the study endpoint (day 14), in accordance with AVMA guidelines for euthanasia.

#### 2.7.2 H&E and AB-PAS staining

The distant colon was fixed with paraformaldehyde, embedded in paraffin, sliced into thin section with thickness of 5 μm and dewaxed for hematoxylin-eosin (H&E) or Alcian Blue-Schiff Periodate (AB-PAS) staining. The morphology of colon was imaged by optical microscope (80i, Nikon, Japan). Histological morphology was scored by inflammatory cells infiltration, the mucosa edema and epithelial crypt loss according to the protocol in previous study.

#### 2.7.3 The IHC staining of IL-6, TNF-α, IL-1β and MCP-1

Colonic tissue specimens were cut into 5-μm-thick sections, which were then blocked with bovine serum albumin (BSA), followed by overnight incubation at 4 °C with primary antibodies against the following targets: rabbit polyclonal IL-1β (1300, Abcam®), mouse monoclonal TNF-α (1,200, Santa Cruz®), rabbit polyclonal IL-6 (1300, Affinity®), and rabbit polyclonal MCP-1 (1400, Abcam®). After three washes with PBST, the sections were exposed to horseradish peroxidase (HRP)-conjugated secondary antibodies (goat anti-rabbit IgG H&L or goat anti-mouse IgG H&L) for 1 h at 37 °C. Following thorough rinsing, antibody binding was visualized using a DAB chromogen kit (ZSGB-BIO, Beijing, China). Finally, the stained sections were observed under a Nikon ECLIPSE 80i microscope (Nikon, Japan) at various magnifications.

#### 2.7.4 Masson's trichrome and Sirius red staining

To further assess colonic fibrosis, collagen deposition in colon tissues was evaluated using Masson's trichrome and Sirius red staining. Paraffin-embedded colonic sections were stained with a Sirius red kit (Solarbio, China) following the manufacturer's instructions. Stained sections were then visualized under a Nikon ECLIPSE 80i microscope (Nikon, Japan) at 400 × magnification.

#### 2.7.5 Immunofluorescence staining of ZO-1, Claudin-5, Occludin-1

After dewaxing, colonic sections were treated with hydrogen peroxide to block endogenous peroxidase activity, followed by antigen retrieval and blocking with BSA. The sections were then sequentially incubated with primary and secondary antibodies. The primary antibodies used were as follows: rabbit polyclonal anti-ZO-1 (1:100, Abcam®), rabbit polyclonal anti-Occludin-1 (1:100, Abcam®), anti-Claudin-5 (1:100, Abcam®), and rabbit polyclonal anti-β-actin (1200, Abcam®). Subsequently, the sections were exposed to Alexa Fluor-conjugated secondary antibodies: goat anti-rabbit IgG Alexa Fluor 647 (1:1000, Abcam®) or donkey anti-rabbit IgG Alexa Fluor 488 (11,000, Abcam®). Nuclei were counterstained with DAPI. Fluorescence images were captured using an inverted fluorescence microscope (Nikon TI-S, Japan).

### 2.8 Statistical analysis

GraphPad Prism 9 (GraphPad Software, USA) was used for all statistical analyses. Quantitative data are presented as mean ± standard deviation (SD) derived from a minimum of three independent replicates. Comparisons between two groups were performed using Student's *t*-test, while comparisons involving multiple groups were evaluated by one-way analysis of variance (ANOVA). Each experiment was repeated three times. Statistical significance was defined as *p < 0.05, **p < 0.01, and *p < 0.001.

## 3 Results and discussion

### 3.1 Characteristics of PG oils

**Fig. 2:**
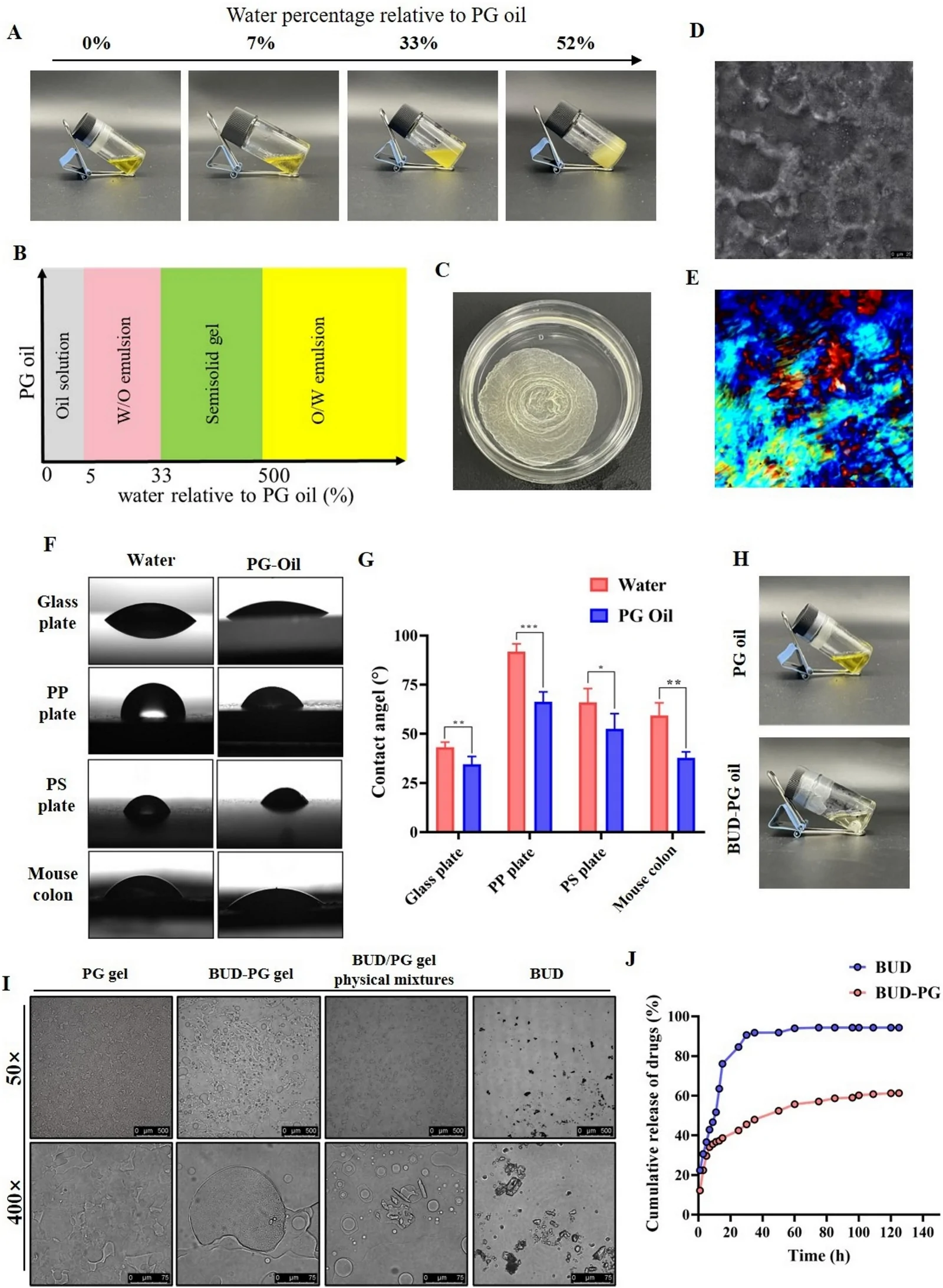
Characterization of water-responsive PG oil: (A) macroscopic appearance of PG oil in presence of water; (B) phase diagram of PG/Water mixture; (C) PG gel-like film on water interfaces; (D) intrinsic morphology of PG gel-like film under CLSM; (E) intrinsic morphology of PG gel-like film under polarized microscopy; (F)) contact angles of PG oil on various substrates including wet glass plate, polypropylene(PP) plate, Polystyrene (PS) plate and fresh mouse colon; (G) the quantitative analysis of contact angles; (H) the macroscopical photographs of PG oil and BUD-PG oil with 35 mg/mL BUD; (I) microscopic images of PG gel, BUD-PG gel, BUD/PG gel physical mixture and BUS suspension; (J) in vitro release of BUD from BUD suspension (BUD) or BUD-PG gel (*n* = 3, ***p < 0.001, *p < 0.05).

As a key component, PC-98 was completely dissolved in the mixed solvents composed of GDO/ethanol/propylene glycol, presenting a viscous, clear and yellow oil-like liquid (PG oil). The water-responsive gelling was observed for PG oil. As shown in Fig. 2A, the morphology of PG oil transited from clear solution to semisolid gel as the percentage of water inside increased. Based on the water percentage and its dispersion inside, the PG/water system gone through various phage zone, including oil-like solution, W/O emulsion, semisolid gel and O/W emulsion (Fig. 2B). When the water percentage of water relative to PG oil was in range from 33% to 500%, the semisolid gel was formed for PG/water system. The water-responsive gelling of PG oil was greatly dominated by self-assembly of phospholipids. It was previously found that at least 20% of PC-98 in PG oil formula was required for its sol-gel transition (Zhao et al., 2023). Moreover, when PG oil drops gently flatten and spread out on water interfaces, the robust gel-like film was formed (Fig. 2C). By contrast, the brittle precipitates were observed for PC-98/ethanol solution without GDO, indicating GDO was key for gel-like film formation. The intrinsic morphology of PG film was further observed by CLSM and results was shown in Fig. 2D. PG film presented the crosslinked networks with the mesh-like shape. Moreover, the optical anisotropy of PG film was presented the polarized microscopy, indicating the unique characteristic of lamellar liquid crystalline (Fig. 2E). Laminated liquid crystals might be a paramount reason why PG film had robust texture. The wetting of PG oil against various substrates was further evaluated by contact angles test. As shown in Fig. 2F/G, PG oil presented the larger contact angles on these substrates than that of water, indicating its good wetting and spreading properties. This might be due to the amphiphilic characteristic of PC-98 and its water-responsive self-assembly on the biologic interfaces. In order to confirm whether PG oil undergoes the phase transition under actual colonic conditions, we further infused PG oil into simulated colonic fluid (SCF) to observe its gelling process. As shown in Fig. S1A, PG oil also undergoes the rapid phase transition in a physiologically relevant medium. Besides, the gelling process was also observed on fresh pig colon (Fig. S1B). To directly evaluate the bio-adhesive properties of PG gel on colonic mucosa, the adhesive strength test and flushing resistance test was further performed. As shown in Fig. S2A, PG gel could sustain 30 g of weight. Moreover, PG gel remains firmly attached to colonic mucosa even after vigorous water washing, whereas a non-gelling control is largely washed away (Fig. S2B/C). These results indicated the good bio-adhesive properties of PG on colonic mucosa. Budesonide (BUD) is a glucocorticoid with potent anti-inflammatory activity but low systemic efficacy because of low solubility and rapid diversion (Miehlke et al., 2018; Cristelo et al., 2025). The solubility of BUD in water was as low as 0.021 mg/mL, while it was easily dissolved in PG oils and its solubility was reaching to 35 mg/mL (Fig. S3A). Moreover, BUD concentration in the saturated solution was not significantly changed after 7 days (Fig. S3B), indicating its equilibrium solubility rather than supersaturation or colloidal dispersion. BUD was dissolved in PG oil at 35 mg/mL to prepare BUD-PG oil for the following experiment. As shown in Fig. 2H, the white BUD powder was easily dissolved in PG oil, forming clear and light-colored solution. To confirm the dispersing status of BUD after gelling BUD-PG oil, the morphology of BUD-PG gel was further imaged by optical microscopy. As shown in Fig. 2I, there existed no crystal granule of BUD in sight for BUD-PG gel. This suggested that BUD was uniformly encapsulated in lamellar liquid crystalline of PG gel. In vitro release of BUD from BUD-PG oil was further detected and results were shown in Fig. 2J. BUD was rapidly released from BUD suspension within 48 h in manner of dissolution-dominated mechanism, while the sustained-release profile of BUD was presented for BUD-PG oil. This might due to the fact that BUD was encapsulated in lamellar liquid crystalline of PG gel.

### 3.2 The therapeutic effect of BUD-PG on colitis mice

**Fig. 3:**
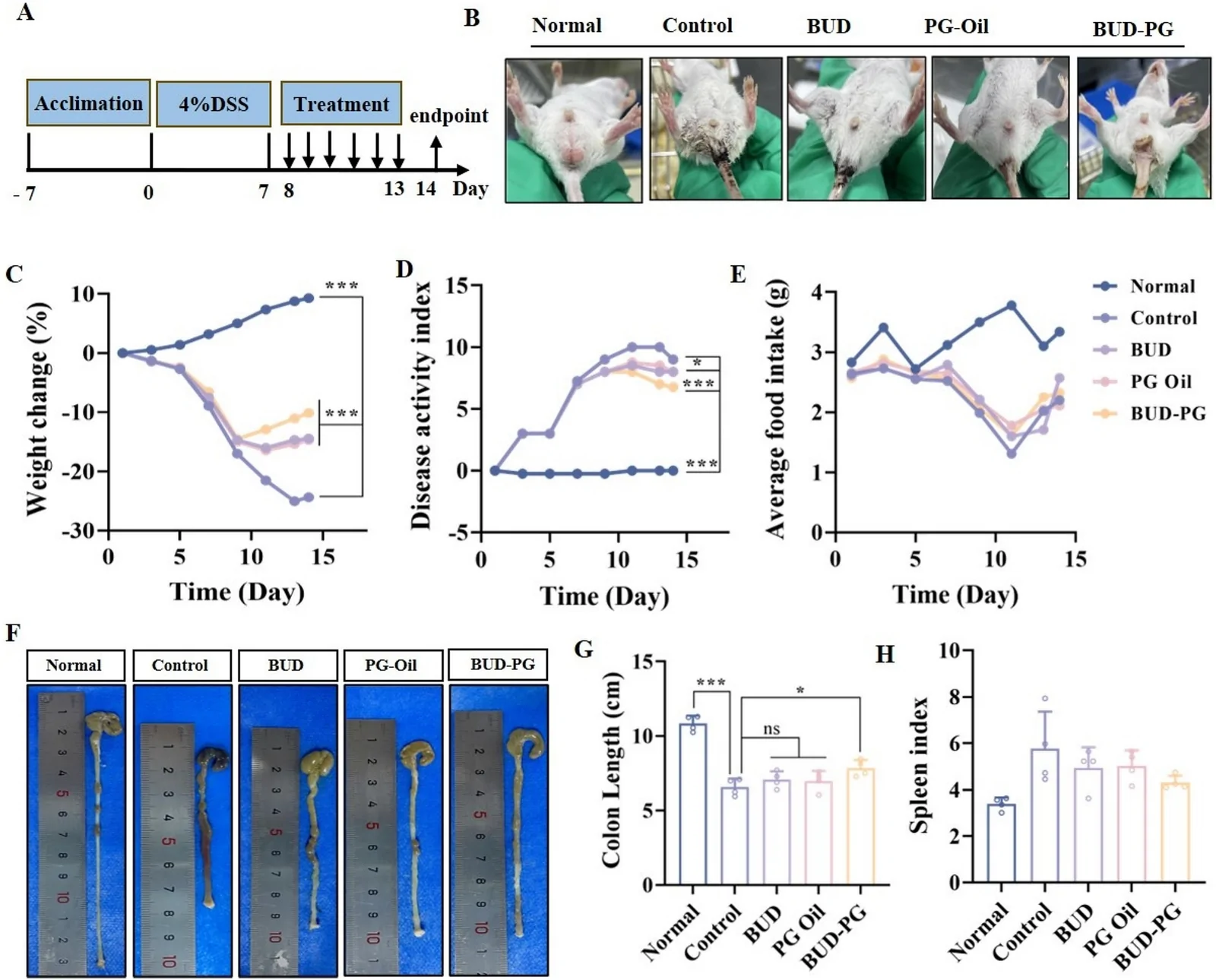
In vivo therapeutic effect of BUD-PG oil on DSS-induced colitis mice: (A) the experimental regime of BUD-PG treatment; (B) Stool status of colitis mice on 14th day after treatment; (C) body weight, (D) DAI scores and (E) Average daily food intake for the colitis mice treated with different enemas (***p < 0.001, *p < 0.05, multiple groups comparisons for endpoint measurements was performed by one-way ANOVA plus Tukey's HSD post hoc test); (F) the macroscopic colons and (G) the statistic of colonic length after treatments; (H) the spleen index from colitis mice (***p < 0.001, *p < 0.05).

To evaluate the therapeutic potential of the water-responsive PG oil formulation encapsulating budesonide (BUD-PG), DSS-induced colitis mice were treated daily via enema for 6 days with free budesonide (BUD), the PG carrier alone, or BUD-PG at doses of 0.3 mg/kg BUD-PG. Free budesonide suspension as positive control was intentional at this proof-of-concept stage to establish the baseline therapeutic activity of the drug and to isolate the specific contributions of the PG oil delivery system. This approach allows for unambiguous attribution of enhanced efficacy to the water-responsive gelling and bio-adhesive properties of PG oil. The experimental regime was presented in Fig. 3A. Disease severity was assessed through multiple parameters, including bodyweight, disease activity index (DAI), food intake, colon length, food intake, and spleen index (Alqudah et al., 2025). DSS-induced colitis presented the obvious stool consistency and occult blood, which was greatly ameliorated by BUD-PG treatment (Fig. 3B). DSS-induced colitis mice displayed the obviously decrease in body weight, which was also significantly alleviated after treatments with Free BUD, PG oil or BUD-PG (Fig. 3C). Average daily food intake was significantly decreased in control mice compared to normal mice (3.34 g) (Fig. 3D). Free BUD partially restored food intake, while BUD-PG normalized food intake, indicating improved overall health status and reduced systemic illness behavior. The Disease Activity Index (DAI), a composite score of weight loss, stool consistency, and occult blood, was elevated in control mice (9.0 ± 0.8) and remained high in the Oil group or Free BUD (Fig. 3D). Moreover, the direct head-to-head comparison between BUD-PG and free BUD revealed substantial therapeutic superiority of the in-situ gelling formulation. BUD-PG restored body weight to 89.9 ± 0.6% of baseline, representing a 4.4% improvement over free BUD (85.5 ± 0.4%) (Fig. 3A). The DAI score in BUD-PG-treated mice (6.7 ± 0.5) was reduced by 16.3% compared to free BUD (8, *p* < 0.01). Besides, the colon of colitis mice in each group was collected at the observation endpoint for macroscopic observation. As shown in Fig. 3F, the colon in DSS-induced colitis mice was obviously shorten and swollen in comparison with that in normal group. Administration of PG carrier alone did not improve colon length (7.0 ± 0.6 cm, *p* > 0.05 vs. control). Colon length recovery was enhanced by 9.9% with BUD-PG (7.8 ± 0.5 cm) compared to free BUD (7.1 ± 0.5 cm), while the spleen index was reduced by 12.2% (4.3 ± 0.3 vs. 4.9 ± 0.8). These findings demonstrate that the PG oil formulation not only improves drug delivery but also significantly amplifies the therapeutic response at an equivalent drug dose.

### 3.3 Alleviating the inflammatory response

The excessive accumulation of inflammatory cytokines was typical indicators of colitis flaring up.

**Fig. 4:**
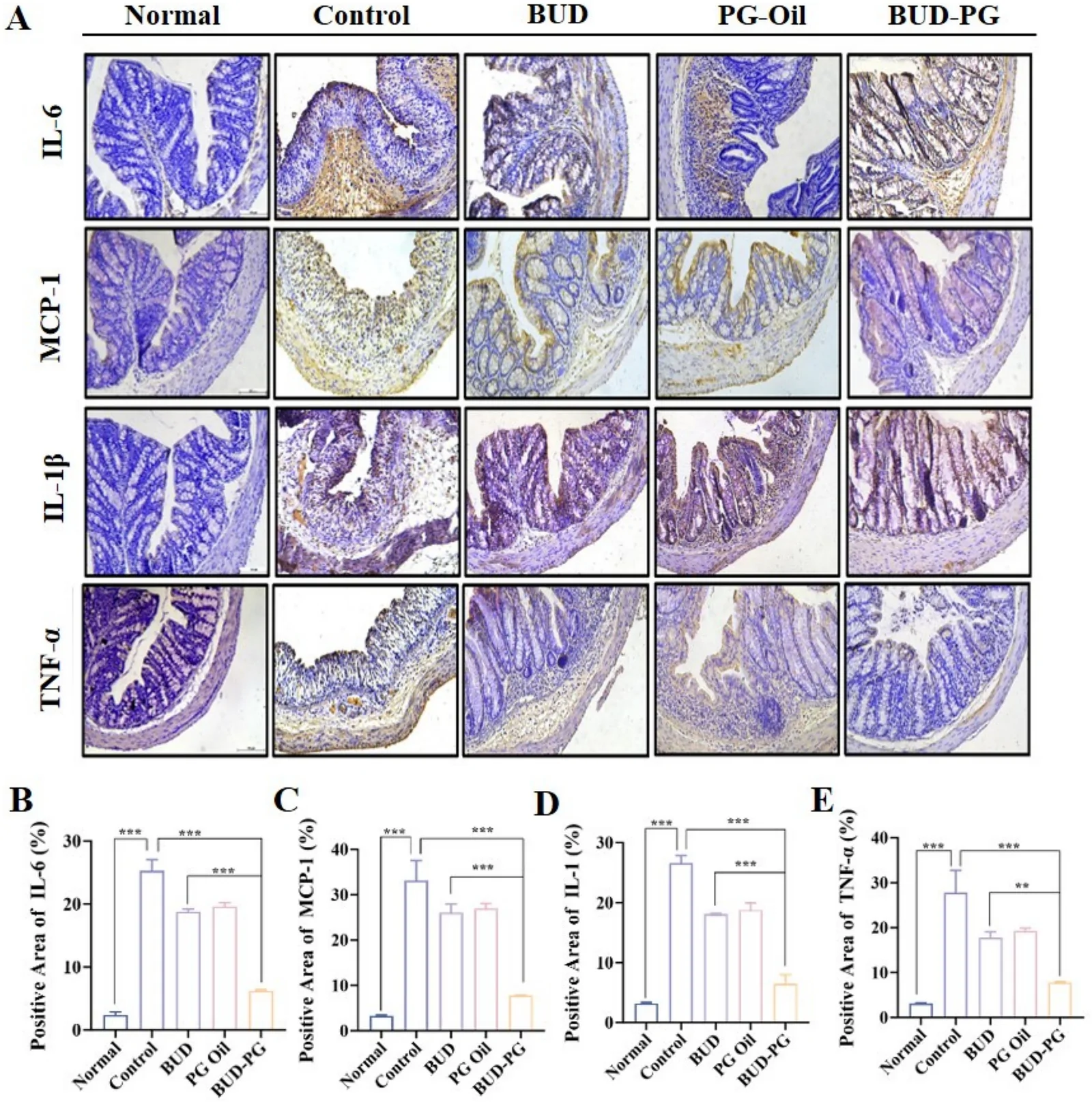
BUD-PG alleviated the inflammatory response: (A) IHC staining of IL-6, TNF-α, IL-1β and MCP-1; the quantitative assay of (B) IL-6, (C) MCP-1, (D) IL-1β and (E) TNF-α based on IHC staining (Data are given as means ± SEM; ^∗∗∗^*p* < 0.001, ^∗∗^*p* < 0.01, ^∗^*p* < 0.05).

Immunohistochemical analysis of pro-inflammatory mediators (IL-6, MCP-1, IL-1β, and TNF-α) was performed to evaluate the alleviating effect of BUD-PG on inflammatory response (Cristelo et al., 2025; Fan et al., 2024). These proinflammatory cytokines were obviously overexpressed in gut epithelial mucosa of DSS colitis mice (Fig. 4A) (Lin et al., 2025). By contrast, their expressions were substantially alleviated by PG, BUD or BUD-PG treatment (Fig. 4B-E). In control mice, robust upregulation of all four mediators was evident, reflecting the severe inflammatory state induced by DSS (Yang et al., 2025). In control group, IL-6 (25.3 ± 1.7%), MCP-1 (33.1 ± 4.4%), IL-1β (26.6 ± 1.2%), and TNF-α (27.7 ± 4.9%) were all markedly elevated compared to normal mice (2.4 ± 0.5%, *p* < 0.001). The magnitude of anti-inflammatory enhancement achieved by BUD-PG over free BUD was particularly striking. BUD-PG reduced IL-6 expression by 66.8% compared to free BUD (6.2 ± 1.7% vs. 18.7 ± 0.5%, *p* < 0.001), TNF-α by 56.4% (7.8 ± 1.8% vs. 17.9 ± 1.2%, *p* < 0.01), IL-1β by 64.1% (6.5 ± 1.5% vs.18.11 ± 0.1%, *p* < 0.001), and MCP-1 by 70.1% (7.8 ± 0.1% vs. 26.1 ± 1.9%, *p* < 0.001). Notably, BUD-PG achieved near-complete normalization of all four inflammatory markers to levels comparable to healthy controls (2.4–3.5%), whereas free BUD only partially suppressed these mediators (17.9–26.1%). This pronounced difference underscores the ability of the PG oil platform to sustain high local budesonide concentrations at the inflamed mucosa, effectively breaking the feed-forward inflammatory cascade that perpetuates colitis. IL-6 drives T-cell resistance to apoptosis and perpetuates chronic inflammation; IL-1β activates NF-κB and amplifies the inflammatory cascade; MCP-1 recruits monocytes and macrophages to the inflamed site; and TNF-α serves as a surrogate for TNF-α signaling, a master regulator of intestinal inflammation (Schneider et al., 2010). The ability of BUD-PG to simultaneously suppress these pathways reflects the sustained, high-concentration exposure of budesonide to the inflamed mucosa enabled by the water-responsive PG formulation (Shabana et al., 2024). Upon rectal administration, BUD-PG oil responded to water and undergone a phase transition to form a bio-adhesive, in situ gelling depot. This prolongs mucosal retention and sustains drug release, thereby maintaining therapeutic budesonide concentrations at the target site over an extended period, in contrast to free budesonide which is rapidly cleared (Watanabe et al., 2024). The resulting sustained suppression of the inflammatory cascade not only accounts for the superior clinical and histological outcomes but also explains the normalization of systemic markers such as spleen index, as the robust local control of inflammation abrogates the systemic spillover of inflammatory cells and cytokines (Yang et al., 2026). Collectively, these molecular findings provide mechanistic validation for the superior efficacy of BUD-PG, demonstrating that the water-responsive formulation achieves true mucosal healing by sustaining high local concentrations of budesonide to comprehensively suppress the core inflammatory pathways driving colitis pathogenesis.

### 3.4 Reduction of collagen deposition and improvement of colonic architecture

**Fig. 5:**
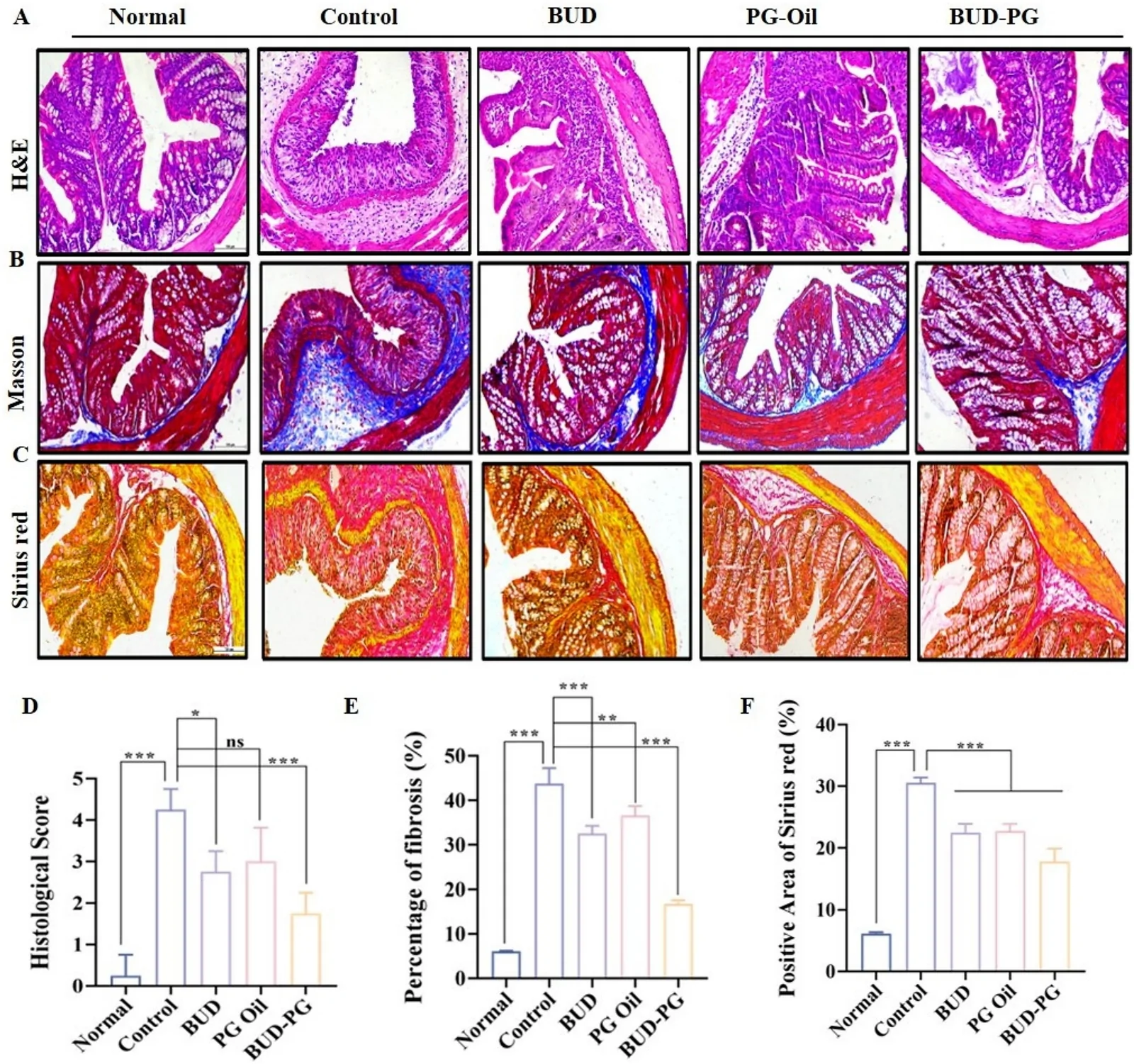
BUD-PG promoted the non-fibrotic repair of colon morphology of colitis mice: (A) H&E staining; (B) Masson's trichrome staining; (C) Sirius red staining; (D—F) the histological score based on H&E, Masson's trichrome and Sirius red staining (Data are given as means ± SEM; ^∗∗∗^*p* < 0.001, ^∗∗^*p* < 0.01, ^∗^*p* < 0.05). (For interpretation of the references to colour in this figure legend, the reader is referred to the web version of this article.)

To further evaluate the quality of mucosal healing beyond macroscopic and clinical parameters, colonic tissue sections were subjected to histological analysis using hematoxylin and eosin (H&E) staining for overall architecture and inflammation, Masson's trichrome staining for collagen deposition (fibrosis), and Sirius red staining for collagen organization and maturation (Kurashima and Kiyono, 2017). As shown in Fig. 5A, the epithelial or crypt was severely destroyed and the massive neutrophils was extensively infiltrated in the lamina propria in DSS group. Besides, the obvious edema was observed in colon submucosa of DSS colitis mice (Xian et al., 2025). By contrast, these pathological indicators were greatly repaired by BUD-PG treatments. H&*E*-stained sections were evaluated using a validated histological scoring system assessing inflammatory cell infiltration, crypt damage, and mucosal architecture. As shown in Fig. 5D, the DSS control group exhibited a high histological score (4.3 ± 0.5), reflecting severe inflammation, crypt loss, and epithelial disruption. Administration of the PG carrier had the slight improvement in histology (3.0 ± 0.8, *p* > 0.05 vs. control), confirming its intrinsic protection toward colitis mice. Treatment with free BUD modestly reduced the histological score to 2.7 ± 0.5 (*p* < 0.05 vs. control). In contrast, BUD-PG significantly reduced the histological score to 1.7 ± 0.5 (*p* < 0.001 vs. control), indicating their effective restoration of colonic architecture.

Masson's trichrome staining was employed to quantify collagen deposition, which typically follows chronic inflammation and indicates pathological tissue fibrotic remodeling (Ye et al., 2025). Representative images (Fig. 5B) revealed extensive blue-stained collagen deposition in the colonic submucosa and muscularis of control mice. Quantitative analysis (Fig. 5E) showed that the percentage of collagen-positive area was significantly elevated in control mice (43.8 ± 3.4%) compared to normal mice (6.1 ± 0.1%, *p* < 0.001). BUD-PG treatment significantly reduced the collagen-positive area compared to free BUD (16.7 ± 0.9% vs. 32.5 ± 1.7%, *p* < 0.001), achieving a level of collagen deposition approaching that of healthy tissue (6.1 ± 0.1%). Sirius red staining under polarized light allows differentiation between mature, densely packed collagen fibers (birefringent red-orange) and immature, loosely packed collagen (green-yellow), providing insight into the quality of tissue repair (Zeng et al., 2025). Representative images (Fig. 5C) showed that control mice exhibited abundant mature collagen deposition. Similarly, Sirius red staining demonstrated a 20.9% reduction in collagen-positive area with BUD-PG versus free BUD (17.8 ± 2.1% vs. 22.5 ± 1.4%, *p* < 0.001) (Fig. 5F). The substantial reduction in collagen accumulation observed with BUD-PG treatment supports its potential to promote favorable mucosal healing. Nevertheless, DSS-induced colitis model used in this study is relatively short-term (14 days), and definitive assessment of anti-fibrotic effect would require additional markers such as α-SMA, collagen I/III ratio, TGF-β/Smad signaling, and hydroxyproline content analysis in future studies.

### 3.5 Repairing gut mucus and epithelial tight junction

**Fig. 6:**
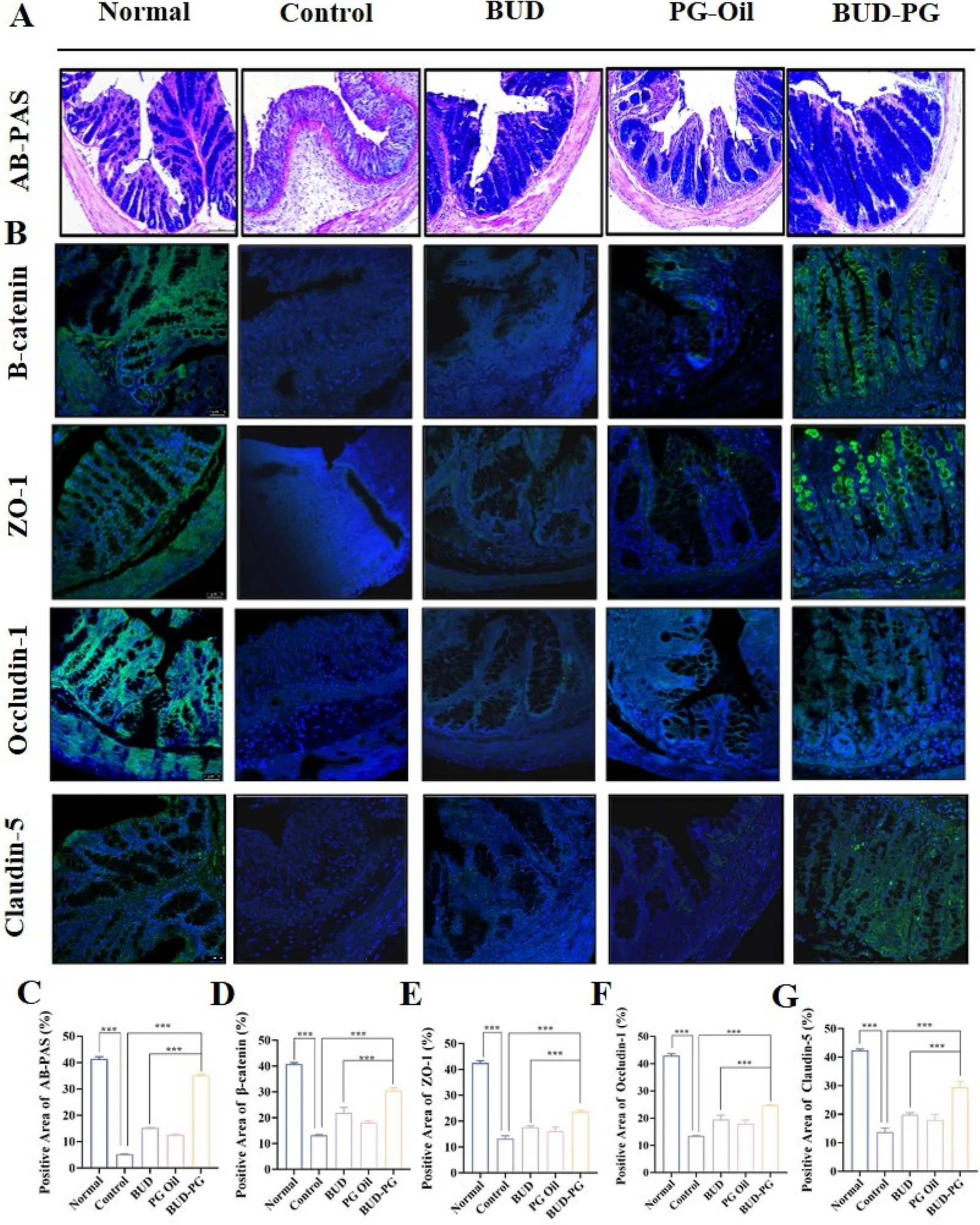
BUD-PG repaired gut mucus and epithelial tight junction: (A) AB-PAS staining of the glycosylated mucus and (B) immunofluorescence staining of β-catenin, claudins (Claudin-5), Zona Occludens-1 (ZO-1), and Occluding-1; the quantitative assay of (C) mucus-producing goblet cells based on AB-PAS staining, (D) β-catenin, (E) ZO-1, (F) Occluding-1 and (G) Claudin-5 based on immunofluorescence staining (Data are given as means ± SEM; ^∗∗∗^*p* < 0.001, ^∗∗^*p* < 0.01, ^∗^*p* < 0.05).

Gut barrier composed of mucus and epithelial tight junction play important roles in preventing translocation of microbial components into the body. Disorders of gut barrier was highly associated with pathology of colitis (Selvakumar and Samsudin, 2025). Mucus is produced by goblet cells and Alcian Blue Periodic acid Schiff (AB-PAS) staining has been performed to quantify the number of goblet cells (Nie et al., 2025). To assess the impact of BUD-PG on intestinal barrier integrity, colonic tissues were evaluated using AB-PAS staining for mucus-producing goblet cells. Compared to the normal group, goblet cells in the DSS group were markedly depleted. By contrast, BUD, PG or BUD-PG treatment led to the obvious recovery of goblet cells (Fig. 6A). Moreover, the gut barrier-restoring capacity of BUD-PG was markedly superior to that of free BUD (Fig. 6A/F). Goblet cell density, a surrogate for mucus production, was restored 56.6% more effectively by BUD-PG (35.2 ± 0.4% cells/field) compared to free BUD (15.3 ± 0.1%, *p* < 0.001). Gut barrier integrity is maintained by the tight junction proteins such as β-catenin, claudins (CLDN), Zona Occludens-1 (ZO-1), and Occluding-1 that are critical for epithelial cell barrier functions. The expression of these tight junction proteins was further detected by immunohistochemistry staining (Wang et al., 2025; Sridhar et al., 2025). As shown in Fig. 6B, these proteins were disordered along the inner lining of the columnar epithelium of the colon tissue, and the expression levels of their expression were substantially decreased in DSS-induced colitis mice (Hu et al., 2025). These deficits underlie the increased paracellular permeability, bacterial translocation, and amplification of inflammation characteristic of active colitis. Tight junction protein expression showed similarly enhanced recovery after BUD-PG treatment: ZO-1 expression was increased by 33.3% (23.6 ± 0.6% vs. 17.7 ± 0.4%, *p* < 0.001), Occludin-1 by 27.2% (24.8 ± 0.1% vs. 19.5 ± 1.6%, *p* < 0.001), and Claudin-5 by 49.0% (29.5 ± 2% vs. 19.8 ± 0.7%, *p* < 0.001) compared to free BUD. The restoration of β-catenin, a key component of adherence junctions and a regulator of Wnt signaling is particularly significant, as it indicates not only recovery of cell-cell adhesion but also re-establishment of the crypt progenitor niche required for ongoing epithelial renewal (Dong et al., 2022). Furthermore, the coordinated restoration of multiple tight junction proteins (ZO-1, Occludin-1, Claudin-5) suggests that BUD-PG achieves functional barrier closure (Chen et al., 2025; Shi et al., 2023). To directly evaluate functional barrier integrity, we performed an in vivo FITC-dextran intestinal permeability assay. As shown in Fig. S4, DSS-induced colitis mice exhibited significantly elevated serum FITC-dextran fluorescence, indicating increased intestinal permeability. BUD-PG treatment substantially reduced serum FITC-dextran levels compared to free BUD or control groups. The extent of barrier restoration correlated with the upregulation of tight junction proteins observed by immunofluorescence. Collectively, these findings establish that BUD-PG not only suppresses inflammation but also actively restores the structural and functional integrity of the gut barrier.

### 3.6 Safety of BUD-PG oil

To evaluate the systemic safety of BUD-PG oil after repeated rectal administration, we have performed histological examination of major organs (heart, liver, spleen, lung, and kidney) from all treatment groups, in addition to the colonic histology already presented. As shown in Fig. S5, no obvious pathological abnormalities were observed in the heart, liver, spleen, lung, or kidney of any treatment group compared to normal controls. Besides, no evidence of organ toxicity, inflammation, necrosis, or structural damage was detected in any of the major organs. These indicated that neither the PG oil vehicle nor BUD-PG formulation caused systemic toxicity after repeated rectal administration.

## 4 Conclusion

In this study, we successfully developed a water-responsive in situ phase-change phospholipid oil (PG oil) as an effective rectal delivery platform for budesonide (BUD), a poorly water-soluble corticosteroid, to treat ulcerative colitis. The PG oil formulation, composed of soybean phosphatidylcholine (PC-98), glyceryl dioleate (GDO), propylene glycol, and anhydrous ethanol, exhibited excellent drug solubilizing capacity for BUD (35 mg/mL) compared to its negligible aqueous solubility (0.021 mg/mL). Upon contact with colonic fluid, the PG oil rapidly underwent a sol-gel transition, forming a robust, bioadhesive lamellar liquid crystalline gel matrix that tightly adhered to the colonic mucosal surface. This gel film not only served as a physical barrier to protect the damaged gut epithelium but also as a sustained-release depot for BUD. In vitro release studies demonstrated that BUD-PG oil provided sustained drug release over 48 h, in contrast to the rapid dissolution-dominated release of free BUD suspension. In a DSS-induced mouse model of colitis, rectal administration of BUD-PG oil significantly outperformed free BUD suspension across multiple therapeutic endpoints. BUD-PG treatment effectively restored body weight, normalized the disease activity index, improved daily food intake, reversed colon shortening, and reduced spleen index to near-normal levels. Mechanistically, BUD-PG dramatically suppressed the overexpression of key pro-inflammatory cytokines (IL-6, TNF-α, IL-1β, and MCP-1) in colonic tissue, indicating potent local anti-inflammatory activity. Histological evaluation revealed that BUD-PG not only repaired epithelial and crypt architecture but also prevented pathological collagen deposition. Furthermore, BUD-PG replenished mucus-producing goblet cells and re-established tight junction proteins (ZO-1, Occludin-1, Claudin-5) and adherens junction protein (β-catenin), which are essential for maintaining paracellular sealing and preventing bacterial translocation (Saha et al., 2025). The therapeutic efficacy of BUD-PG oil arises from the complementary and synergistic actions of its two components. The combination of BUD and PG oil achieves comprehensive mucosal healing that neither component alone can fully accomplish. Collectively, BUD-PG formulation represents a promising, simple-to-prepare, and effective therapeutic strategy for ulcerative colitis management, with the potential to achieve sustained remission. The translational potential of any novel drug delivery platform depends critically on the safety and regulatory status of its constituent excipients, particularly for the intended route of administration. PC-98 used in our formulation is of high purity (≥98%) and derived from soybean, a GRAS (Generally Recognized as Safe) source, which supports its acceptability as a pharmaceutical excipient. Although GDO as a safe ingredient has been listed as the Cosmetic Ingredient Review (CIR) Expert Panel, our formulation uses GDO at a concentration (50% *w*/w) that is higher than typical cosmetic use levels. This higher concentration reflects the formulation's unique function as an in-situ gelling matrix, and it would necessitate additional toxicological evaluation specific to the rectal route in future. Budesonide rectal foam products (e.g.*,* Uceris®) represent the current standard for localized budesonide delivery in distal ulcerative colitis. Future investigations will directly compare BUD-PG oil against foam products in the DSS-induced colitis model, evaluating clinical endpoints, pharmacokinetic profiles, and mucosal healing outcomes.

## CRediT authorship contribution statement

**Ting Ouyang:** Writing – original draft, Investigation, Conceptualization. **Yumo Chen:** Methodology, Investigation. **Yiying Jia:** Methodology, Investigation. **Jiarui Li:** Methodology, Investigation. **Zhouyang Tan:** Methodology. **Kaili Lu:** Methodology, Investigation. **Minmin Wang:** Methodology. **Helin Xu:** Writing – review & editing, Supervision, Funding acquisition. **Lifen Wang:** Writing – review & editing, Resources, Funding acquisition.

## Ethics declaration

This study was conducted in accordance with the ARRIVE (Animal Research: Reporting of In Vivo Experiments) guidelines.

This study was approved by the Institutional Animal Committee.

(Approval No. xmsq 2024–0281).

## Declaration of competing interest

The authors declare that they have no known competing financial interests or personal relationships that could have appeared to influence the work reported in this paper entitled: *“Water-triggered in-situ gelling phospholipid oil enema for enhanced budesonide delivery and mucosal healing in ulcerative colitis”*.

All funding sources are acknowledged in the manuscript. No author has any conflict of interest, including but not limited to: employment, consultancies, stock ownership, honoraria, paid expert testimony, patent applications/registrations, or grants/other funding that may be affected by the publication of this work.

## Data Availability

All relevant data are within the paper.
