# Supplementary material for: Water-triggered in-situ gelling phospholipid oil enema for enhanced budesonide delivery and mucosal healing in ulcerative colitis
Source: Int J Pharm X. 2026 Aug 21;12:100647. doi: 10.1016/j.ijpx.2026.100647 (PMC13524639; doi:10.1016/j.ijpx.2026.100647)
Supplement: Supplementary file 1 [file mmc1.docx]

Supporting information

Fig S1 the in-situ gelation process of PG oil in a physiologically relevant medium: (A) the gelling of PG oil in simulated colonic fluid (SCF); (B) the gelling of PG oil on fresh pig colon mucosa.

Fig S2 the adhesive test of PG gel toward fresh mouse colon mucosa: (A) the peeling test of PG gel toward fresh mouse colon; (B) the scheme of flushing test; (C) the remaining PG gel on colon mucosa after vigorous water washing (flow rate of 10 mL/min for 60 seconds; PG gel was labeled with Rhodamine B, and Rhodamine B solution was used as a non-gelling control).

Fig S3 the equilibrium solubility of BUD in PG oil: (A) the equilibrated phase-solubility of BUD in PG oil; (B) the concentration of BUD in saturated solution after 7 days.

Fig S4 FITC-dextran fluorescence intensity in serum on day 14 after treatment

Fig S5 H&E staining of major organs (heart, liver, spleen, lung, and kidney) from all treatment groups.
